# Production of Special Fruit Beer With Addition of Cupuassu (*Theobroma grandiflorum*) Pulp and Prolyl Endopeptidase to Improve Volatile Compounds and Physicochemical Parameters

**DOI:** 10.1111/1750-3841.70916

**Published:** 2026-02-19

**Authors:** Ermor Cesar de Sousa Lopes, Nélio Jacinto Manuel Ualema, Hellen Kempfer Philippsen, Camilo Barroso Teixeira, Stanislau Bogusz Junior, Guilherme Ribeiro da Cunha Nascimento, Gilson C. A. Chagas‐Junior, Braian Saimon Frota da Silva, Nelson Rosa Ferreira

**Affiliations:** ^1^ Laboratory of Biotechnological Processes (LAPROBIO), Graduate Program in Food Science and Technology, Institute of Technology (ITEC), Campus Guamá Federal University of Pará (UFPA) Belém Pará Brazil; ^2^ Department of Agricultural Sciences, School of Agricultural Sciences Save University, Chongoene Maxixe Mozambique; ^3^ Socioenvironmental and Water Resources Institute Federal Rural University of the Amazon (UFRA) Belém Pará Brazil; ^4^ Faculty of Food Engineering (FEA), Institute of Technology (ITEC), Campus Guamá Federal University of Pará (UFPA) Belém Pará Brazil; ^5^ University of São Paulo (USP), São Carlos Institute of Chemistry (IQSC) São Carlos São Paulo Brazil

**Keywords:** Amazon, cupuassu, fermentation, HS‐SPME, PCA, volatile compounds

## Abstract

This study assessed the feasibility of incorporating cupuassu pulp and the commercial protease *Aspergillus niger* prolyl endopeptidase (AN‐PEP) in the production of fruit beer. To preliminarily evaluate the impact of the protease on the beer protein profile, one‐dimensional SDS‐PAGE was employed. The volatile compounds were analyzed using headspace solid‐phase microextraction (HS‐SPME)–GC–MS, whereas physicochemical parameters were evaluated following the American Society of Brewing Chemists (ASBC) methods. The band profiles from electrophoresis indicated the degradation of high‐molecular‐weight proteins, such as gluten, in the formulations treated with protease (A1 and A2). In two out of the three formulations, elevated levels of 3‐methyl‐1‐butanol, linalool, and 2‐phenylethanol were detected, suggesting that the protease may have played a role in the release of volatile flavor compounds. The beers produced exhibited low alcohol content, ranging from 2.80% to 3.44% (v/v). On the basis of these findings, we conclude that the combination of AN‐PEP and cupuassu pulp is a viable approach to producing fruit beer with a predominantly floral, sweet, and whiskey‐like aroma profile, showing preliminary evidence of degradation of high‐molecular‐weight proteins (including potential gluten fractions) by the action of the protease.

**Practical Applications:**

The results obtained in this study serve as a foundation for the development of new approaches in the artisanal and sustainable production of fruit beer, providing information for the creation of innovative beers with added value, as well as promoting and valorizing local ingredients for the craft beer sector.

## 1 Introduction

Beer is one of the most widely consumed beverages in the world, with a history spanning over 8000 years (Cela et al. 2020). The proliferation of specialty malts and hops and the development of yeast genetic engineering, combined with growing interest in new beer styles with distinct sensory stimuli and concerns about health promotion, are leading to continued growth in craft beer production in recent years (Habschied et al. 2020; Baiano 2021; Iattici et al. 2020).

The main innovation trends in craft beer production are related first to the production of functional beer, such as organic beers, made only with organic and genetic modified organism (GMO)‐free ingredients, low‐calorie beer, low‐alcohol beer, nonalcoholic beer, and low‐gluten or gluten‐free beer (GFB), which are produced mainly to meet the search for healthy consumption (Martínez et al. 2017; Cela et al. 2020). The other relates to beer with distinct sensory stimulation, such as special flavors and aromas, achieved through technology like non‐*Saccharomyces* yeasts or the addition of other substances, including herbs, spices, and fruits (Kim et al. 2017; Sampaolesi et al. 2023).

The healthier and more functional beer, with low‐gluten or gluten‐free options, is becoming popular as a response to cater to people suffering from various hypersensitivities or intolerances to gluten (Mickowska et al. 2023). To achieve popularity, many craft breweries employ various strategies, such as brewing with gluten‐free cereals or pseudocereals. Other approaches involve the use of non‐grain materials, genetic engineering to remove toxic epitopes in gluten‐containing cereals, controlled hydrocavitation‐assisted silica gel treatment, and enzymatic treatment (Mickowska et al. 2023; Burdzaki et al. 2024; Cela et al. 2023). However, the use of non‐gluten‐containing cereals in beer production affects the aroma, taste, and other sensory properties of beer.

On the other hand, genetic engineering still needs to be widely accepted, and non‐grain materials are associated with the production of undesirable acid compounds that negatively affect the flavor of the final beer (Gobbi et al. 2024; Burdzaki et al. 2024; Scherf et al. 2018; Cela et al. 2023). Therefore, the use of specific enzymes, such as *Aspergillus niger* prolyl endopeptidase (AN‐PEP) (EC 3.4.21.26), for GFB production is a viable option, as they are food‐grade and compliant with legislation, easily integrate into existing industrial production processes, and exhibit low costs (Cela et al. 2020).

In the sector of beers with a special sensory stimulus, the fruit beer segment is experiencing rapid expansion. This class of specialty beers is made with the addition of fruits/fruit extracts/fruit‐flavored additives to obtain a product with a specific sensory profile, characterized by a clear presence of fruity notes, yet harmonious and balanced with the original base beer (Zhao et al. 2023; Castro Marin et al. 2021).

The addition of whole fruit or its parts, such as peels and pulp, is the most popular option due to its ease of preparation (Zhao et al. 2023). The addition of fruit allows for the use of local raw materials and the incorporation of regional flavors into craft beer. Cupuassu (*Theobroma grandiflorum*) is an important fruit from the Amazon region and was used in this study for the peculiar flavor and aroma due to its characteristic, intense aroma and pleasantly acidic flavor in its pulp. Cupuassu has already been used as an adjunct in fruit–wheat beer, resulting in a reduction in bitterness and an improvement in the aroma of the final product (Ipiranga et al. 2022).

In this context, two significant trends currently dominate the development of craft and specialty beers (Zhao et al. 2023; Baiano 2021). The first encompasses functional beers, developed to meet the demand for products with low‐calorie, nonalcoholic, or low‐alcohol content, and, above all, with low or no gluten content (Mickowska et al. 2023; Cela et al. 2020). The second trend reports beers with enhanced or innovative sensory profiles, among which fruit beers stand out as one of the fastest‐growing segments (Zhao et al. 2023; Castro Marin et al. 2021). Although these two strategies have already been investigated in isolation, this study presented a third, unprecedented condition: the effect of AN‐PEP addition on volatile components and physicochemical parameters.

In functional beers, treatment with AN‐PEP is used as an interesting alternative for proteolysis in conventional barley‐based beers, better preserving the typical sensory profile than other approaches used for proteolysis (Cela et al. 2020; Burdzaki et al. 2024). In parallel, the addition of tropical and regional fruits, such as cupuassu, is gaining ground in the fruit beer category due to its pleasant and intense acid‐aromatic profile (Ipiranga et al. 2022).

Therefore, this study systematically investigated the feasibility of adding cupuassu and the commercial protease AN‐PEP to produce a fruit beer with reduced gluten content and a specific aromatic profile. Three beers were produced using cupuassu as an adjunct: one treated with AN‐PEP protease at the beginning of fermentation; one treated with AN‐PEP at the beginning of maturation; and a third, a control without AN‐PEP. A comparison was made between the samples regarding volatile profiles, protein profiles by electrophoresis, and physicochemical parameters.

## 2 Materials and Methods

### 2.1 Treatments

The experimental setup consisted of three treatments (with three independent replicates each) of beers produced with the addition of cupuassu pulp, differing in the timing of incorporation of the protease AN‐PEP. This comparative setup was analyzed using one‐way analysis of variance (ANOVA) followed by Tukey's post hoc test. The protease was added at different stages: A1 (addition at the beginning of fermentation) and A2 (addition at the beginning of maturation). The beer without the addition of protease was considered the control (A3). A summary of the treatments is illustrated in Table 1.

**TABLE 1:** Designation of treatments used for beer production, steps of addition of protease *Aspergillus niger* prolyl endopeptidase (AN‐PEP), repetitions considered, and quantity of beer produced per treatment.

Treatment	Treatment conditions	Repetitions	Quantity of beer produced (L)
**A1**	Addition of AN‐PEP (start of fermentation) + cupuassu	3	3
**A2**	Addition of AN‐PEP (beginning of maturation) + cupuassu	3	3
**A3**	Without the addition of AN‐PEP + cupuassu (control beer)	3	3

A single 27 L batch of wort was brewed, using British golden ale as base beer, and, following cooling and aeration, was divided into three 5 L food‐grade polypropylene, nontoxic, BPA‐free fermenters. Each bucket, equipped with an airtight lid and airlock (with 70% ethanol and ultrapure water), was filled with 3 L of wort. Each treatment (A1, A2, and A3) was then conducted in independent triplicate, totaling nine distinct experimental units.

### 2.2 Mashing and Brewing

A total of 27 L of cupuassu fruit beer was brewed, using 5.5 kg of crushed and sieved Patagonia Pilsen malt and 27.5 L of mineral water sourced from the local market, which were mixed (1:5 w/v) for the mashing program, conducted as follows: 48°C for 30 min, 63°C for 30 min, 72°C for 30 min, and finally, the temperature was raised to 78°C and maintained for 10 min to terminate the reaction and inactivate the amylases. The mixture was filtered to obtain wort, and the spent grain was washed twice at 68°C, first with 10 L and finalized with 4 L. Next, the wort was boiled for 60 min before the whirlpool, with hops (NP cascade from LNF) being added at the beginning of boiling (5 min) and at the end (5 min). The final wort registered 10.5 °P and a pH of 5.4. After cooling, the wort was aerated, and the Ale yeast S‐04 (Fermentis, Marcq‐en‐Baroeul, France) was inoculated with a pitching rate of 10^6^ cells mL^−1^ (Klimczak et al. 2024). Primary fermentation occurred at a temperature of 20°C for 7 days. At the beginning of fermentation, AN‐PEP enzyme (Clarity Ferm, White Labs) was added for A1 treatment. Maturation was held at 4°C for 7 days. At the beginning of this stage, AN‐PEP for A2 treatment was added, as well as cupuassu pulp (purchased pasteurized from the municipal store in Belém city—PA, Brazil—for all investigated beers). During fermentation days, the sugar consumption was monitored by °Brix measurements.

Following maturation, the beers underwent natural carbonation via priming. They were bottled and sealed with standard metal crown caps using a bench capper. The samples then underwent a carbonatation stage for 7 days at 20°C prior to analysis.

### 2.3 Evaluation of Beer Quality Parameters

The following parameters were measured in triplicate in all beer samples: pH, total acidity (TA), total soluble solids (TSS), color, alcohol content by volume (ABV), and bitterness. Prior to analysis, the samples were degassed and all the physicochemical analyses were performed following the American Society of Brewing Chemists (ASBC) protocol (Brewers Association, 2023). This process included immersing the samples in an ultrasonic bath for 15 min (Burdzaki et al. 2024).

The color of degassed samples was assessed by measuring absorbance at 430 nm in a UV‐1600 spectrophotometer (Pró‐Análise, Porto Alegre, Brazil). Values were converted to SRM (×12.7) and EBC (×19.7) units according to ASBC Beer–10A. Results were multiplied by 1.27 to be expressed as SRM and by 1.97 as EBC (Brewers Association, 2023).

To determine the bitterness of the beers, the official ASBC Beer–23A method was employed, which involves extraction with isooctane followed by spectrophotometric analysis at 275 nm (Brewers Association, 2023).

TSS were measured in °Brix using a portable digital refractometer (FG‐11, ATC, USA) and converted to degrees Plato. Original gravity (OG) was determined by a precision hydrometer on the cooled wort before yeast pitching (10.5 °P). Beer‐4C method (Brewers Association, 2023) using the correct Balling formula: real extract (°P) = 0.1808 × OG + 0.8192 × apparent extract (refractometer); ABV (% v/v) = (OG—Real Extract) × 0.1293, and apparent attenuation was calculated as AA% = [(OG—Apparent Extract)/OG] × 100.

### 2.4 CIELab Color Determination

Color determination was carried out using a benchtop spectrophotometer colorimeter (3NH, YS6060, China) with direct reading. The L*, a*, and b* parameters of the CIELab system were measured according to the ASBC analytical method (Brewers Association, 2023) The L* value indicates the position of a point on the vertical axis of luminosity, where it represents lightness or darkness. The a* value indicates the position on the axis corresponding to green for negative values and red for positive values. Similarly, the b* value corresponds to blue (−) and yellow (+) values.

### 2.5 SDS‐PAGE Electrophoresis

One‐dimensional SDS‐PAGE electrophoresis was performed according to the protocol established by Laemmli (1970). In this procedure, 10 µg of proteins were mixed with a buffer containing 0.2 M Tris‐HCl at pH 6.8, 40% (v/v) SDS, 0.1% bromophenol blue, and 20% (v/v) β‐mercaptoethanol. The sample was then heated to 100°C for 2 min and loaded onto a 12% SDS‐PAGE gel (7 cm long and 1.0 mm thick). The electrophoresis was run at 30 V for 30 min, followed by 100 V until completion. To visualize the proteins, the gels were stained with Coomassie blue (GE Healthcare) according to the manufacturer's instructions.

### 2.6 Analysis of the Beer Volatile Compounds (VOCs)

The profile of volatile compounds was determined using a Shimadzu GC–MS 2010 (Shimadzu, Kyoto, Japan), equipped with an electron ionization source and a quadrupole mass analyzer, coupled to a mass spectrometric detector. Sample preparation was performed using headspace solid‐phase microextraction (HS‐SPME), as described by Bogusz et al. (2011). Prior to the identification and quantification of volatile compounds, the extraction conditions were optimized. This process included evaluating different SPME fibers to determine the one with the highest extraction efficiency, establishing strategies for extraction optimization, and ultimately, the separation and identification of the volatile compounds in the samples took place. The conditions for these preliminary steps are described in the Supporting Information S1.

Volatiles were separated using a DB‐5MS fused silica capillary column (30 m × 0.25 mm × 0.25 µm, Agilent Technologies, Santa Clara, CA, USA) under the following chromatographic conditions: The injector was set to 250°C and operated in splitless mode for 1 min, with helium as the carrier gas flowing at 0.6 mL/min. The oven temperature started at 40°C for 1 min, then increased at a rate of 3°C/min until it reached 196°C. Subsequently, the temperature ramped up at 20°C/min to a final temperature of 280°C and was maintained for 2.8 min. The interface temperature was maintained at 280°C, whereas the electron ionization source operated at +70 eV, scanning a mass range from 35 to 350 *m*/*z*. After each extraction and desorption, the SPME fiber was reconditioned at 250°C for 15 min, following the manufacturer's recommendations, to ensure clean blank runs and to prevent carryover effects. All samples were analyzed in triplicate, and the relative standard deviation (RSD%) of the individual volatile compounds and of the total peak area was below 10%, indicating good repeatability and robustness of the HS‐SPME–GC–MS method.

The identification of volatile compounds was achieved by comparing the mass spectra obtained from the sample with those in the NIST 11 library (National Institute of Standards and Technology, USA). A similarity score of at least 85% was required for a match, along with a comparison of the linear temperature‐programmed retention indices (LTPRI), allowing for a maximum variation of ±10 RI units. For calculating the LTPRI, a homologous series of alkanes ranging from C8 to C20 was utilized.

### 2.7 Statistical Analysis of VOCs Results

The data obtained were analyzed using one‐way ANOVA with Statistica 10.0 (StatSoft Inc., OK, USA). A Tukey's test was conducted at a significance level of *p* ≤ 0.05 to identify significant differences between the sample means. Principal component analysis (PCA) and hierarchical cluster analysis (HCA) were employed to investigate the order and relative importance of the three formulations in terms of VOCs using OriginPro 8 software (OriginLab Corporation, Northampton, MA, USA). The Mahalanobis distance was employed to identify residuals with similar volatile compound profiles, using a significance level of 95% with Minitab software version 14.13 (Minitab Inc., State College, PA, USA).

## 3 Results and Discussion

### 3.1 Physicochemical Characterization of Beers

The basic physicochemical properties, pH, TA, color, bitterness, TSS, and ABV of the evaluated beers are presented in Table 2.

**TABLE 2:** Basic physicochemical parameters of investigated Beers A1, A2 and A3.

Physicochemical parameters	A1	A2	A3
pH	3.84 ± 0.01^a^	3.78 ± 0.01^b^	3.71 ± 0.03^c^
Titratable acidity (g/L lactic acid equivalent)	4.26 ± 0.05^a^	4.50 ± 0.01^b^	5.10 ± 0.05^c^
EBC color	20.95 ± 0.77^a^	20.10 ± 0.24^ab^	19.15 ± 0.76^b^
SRM color	10.63 ± 0.39^a^	10.20 ± 0.12^ab^	9.72 ± 0.38^b^
Bitterness (bitter units)	18.60 ± 0.08^a^	15.15 ± 0.02^b^	12.24 ± 0.04^c^
Alcohol ABV (% v/v)	3.44 ± 0.00	3.06 ± 0.00	2.80 ± 0.00
Apparent attenuation (AA%) Apparent extract (°P)	64.29 ± 0.00 3.75 ± 0.05	57.14 ± 0.00 4.5 ± 0.07	52.38 ± 0.00 5.00 ± 0.06

*Note*: pH, titratable acidity (g/L), color SRM/EBC, bitterness (IBU); alcohol by volume (ABV) and apparent attenuation (AA%). Values are mean ± SD (*n* = 3 biological replicates × three technical replicates). Different letters (a–c) on the same line indicate statistical difference (Tukey test, *p* ≤ 0.05).

The pH value ranged from 3.71 to 3.84, thus classifying the beers produced as acidic (Da Silva et al. 2024). The low pH recorded in the beers of our study is justified by the addition of capuassu pulp, which, in its mature stage, presents an acidic characteristic with a pH of around 3–3.68 (Ipiranga et al. 2022; Melo et al. 2021; Da Silva et al. 2024). This condition can be advantageous from the perspective of microbial stability in the beer, as the growth of spoilage microorganisms is inhibited at low pH levels (Gasiński et al. 2020). On the other hand, a low pH can improve and soften the flavor of beers, thus improving drinkability. However, a very low pH can make beers more bitter (Zhao et al. 2023). An approximate pH of 4.06 was recorded in fruit beer with added cupuassu and pitaya (Ipiranga et al. 2022). The pH values in the same range were recorded in mango fruit beer 3.58–3.77 (Gasiński et al. 2020) with passion fruit 3.65–3.95 (Dos Santos et al. 2022) and grapes 3.59–3.77 (Castro Marin et al. 2021). On the other hand, the pH recorded in this study is considered low compared to the pH of conventional beers (4.0–5.5) (Zhao et al. 2023).

TA ranged from 4.26 to 5.10 g/L of lactic acid equivalent, which is considered high and corresponds with the low pH recorded. This result is expected in fruit beers due to the high content and dissolution of organic acids, particularly malic and citric acids, found in fruits like cupuassu. However, these acidity levels exceed those typically found in conventional beers, which range from 1.0 to 1.5 g/L of tartaric acid. Additionally, other fruit beers, such as those made from passion fruit, persimmon, pineapple, and quince, have shown titratable acidity levels ranging from 1.2 to 3.9 g/L (Da Silva et al. 2024; Dos Santos et al. 2022; Costa et al. 2019; Zapata et al. 2019). Therefore, regarding titratable acidity, the beers produced in our study resemble those of the sour style, which can range from 6.0 to 20 g/L (Brewers Association 2023).

The color analysis results indicate that the three formulations produced are classified as slightly dark beers, with color values ranging from 20 to 30 EBC (Strong and England 2015). Beers containing 40% jujube have been recorded with color values in the range of approximately 18.43–33.89 EBC (Yang et al. 2022). In contrast, higher color values of 25.8–41.48 EBC were reported in beers made with added quinces, omija fruits, and red raspberries (Zapata et al. 2019; Deng et al. 2020; Yin et al. 2021). This difference can be attributed to the fact that these fruits have a more intense color compared to cupuassu pulp.

In terms of bitterness results measured by International Bitterness Units (IBU), Sample A1 exhibited the highest bitterness value at 18.60 ± 0.08, whereas the control sample showed the lowest value at 12.24 ± 0.04. These findings indicate that the beers produced are light and smooth, aligning with the styles defined by the Brewers Association: International Pale Lager (18–25 IBU) and American Light Lager (8–12 IBU). The results of our study fall within the range of various commercial beers and some fruit beers documented in the literature, which report IBU values between 8.70 and 21.70 (Zapata et al. 2019; Zhao, Yin, et al. 2023; Yin et al. 2021; Kawa‐Rygielska et al. 2019).

The estimated alcohol content of the final beers ranged from 2.80% to 3.44% ABV, which is considered low compared to most conventional and fruit beers. Typically, fruit beers have a higher alcohol content because the sugars introduced by the fruit during fermentation boost the production of ethanol (Gasiński et al. 2020; Kawa‐Rygielska et al. 2019). The low alcohol content observed resulted from three combined and intentional factors: (i) low initial density (OG = 10.5 °P), aiming for a light and sessionable beer; (ii) addition of cupuassu pulp only at the beginning of maturation (4°C), making most of the fermentable sugars of the fruit unavailable to the yeast during primary fermentation—a strategy adopted to preserve the volatile compounds characteristic of cupuassu (Gasiński et al. 2020; Dos Santos et al. 2022; Ipiranga et al. 2022); and (iii) moderate apparent attenuation (52.4%–64.3%), limited by the very low pH after the addition of the acidic pulp and by the low temperature during maturation.

To increase the ABV in future productions without compromising the sensory profile, it is recommended to: increase the OG of the base wort to 12–13 °P; add 30%–50% of the pulp in the last days of primary fermentation; or use yeasts that are more tolerant to low pH. These changes should allow for a controlled pH of 4.5%–6.0% v/v.

In previous studies, fruit beers with 50% and 75% persimmon had ethanol contents of 4.72% ± 0.15% and 5.63% ± 0.16% (v/v), respectively (Martínez et al. 2017). For cherry, the ethanol content ranged from 4.98% to 5.09% (Kawa‐Rygielska et al. 2019), and for mango, it ranged from 4.13% to 4.63% (Gasiński et al. 2020). Therefore, the low alcohol content observed in our study may appeal to a niche market of consumers who prefer lighter beers.

The significantly higher apparent attenuation observed in Treatment A1 is attributed to the presence of AN‐PEP throughout the entire primary fermentation phase. The proteolytic activity at 20°C released additional assimilable nitrogen (peptides and amino acids), stimulating yeast metabolism and increasing sugar consumption. In contrast, adding the enzyme at the beginning of maturation (A2, 4°C) substantially reduced proteolytic efficiency. At the same time, the control treatment (A3) lacked this additional nitrogen source, explaining the progressively lower attenuation values (64.29%, 57.14%, and 52.38%, respectively).

### 3.2 CIELab Color

As methods based on absorbance (EBC and SRM) have limitations in distinguishing beers with nearly identical EBC or SRM values but different colors, the CIE 1976 L*a*b* color space was incorporated into our research.

The results presented in Table 3 indicate lower L* values, which are closer to 0 than to 100. This suggests that the beers produced have a dark hue, which is consistent with the findings from the EBC absorbance analysis. Conversely, the b* coordinate showed positive values for all samples, indicating that the beers have a yellowish coloration. This outcome can be attributed to the color of the malt used in the beer formulation. The dark hue observed is likely due to the addition of cupuassu pulp, as the inclusion of fruits or their parts typically leads to a decrease in the L* coordinate value (Zhao, Yin, et al. 2023). However, there were no statistically significant differences (*p* > 0.05) among the treatments concerning the L* parameter and the coordinates a* and b*. Notably, Samples A1 and the control exhibited significant differences (*p* ≤ 0.05) when compared to Sample A2.

**TABLE 3:** Average CIE L*a*b* color values of beer A1 (with protease at the beginning of fermentation), A2 (protease at the beginning of maturation), and A3 (control without protease).

Color parameters				
Treatment	L*	a*	b*	Hue angle *H*°
**A1**	29.60 ± 0.08^a^	0.26 ± 0.02^a^	3.60 ± 0.05^a^	85.80 ± 0.26^a^
**A2**	28.42 ± 0.60^b^	−0.16 ± 0.09^b^	2.49 ± 0.72^b^	−85.88 ± 3.00^b^
**A3**	29.29 ± 0.15^ab^	0.25 ± 0.06^a^	3.71 ± 0.05^a^	86.13 ± 1.01^a^

*Note*: Means  ± standard deviation with different lower case letters in the same column are statistically different (Tukey test, *p* ≤ 0.05).

The results recorded in this study agree with those observed in the literature, which indicate that beers produced with pulps, extracts, or parts of fruits as adjuncts tend to present reduced L* values and a consequent dark hue (Koren et al. 2020; Zapata et al. 2019; Castro Marin et al. 2021; Ualema et al. 2024).

### 3.3 Enzymatic Hydrolysis

**FIGURE 1:**
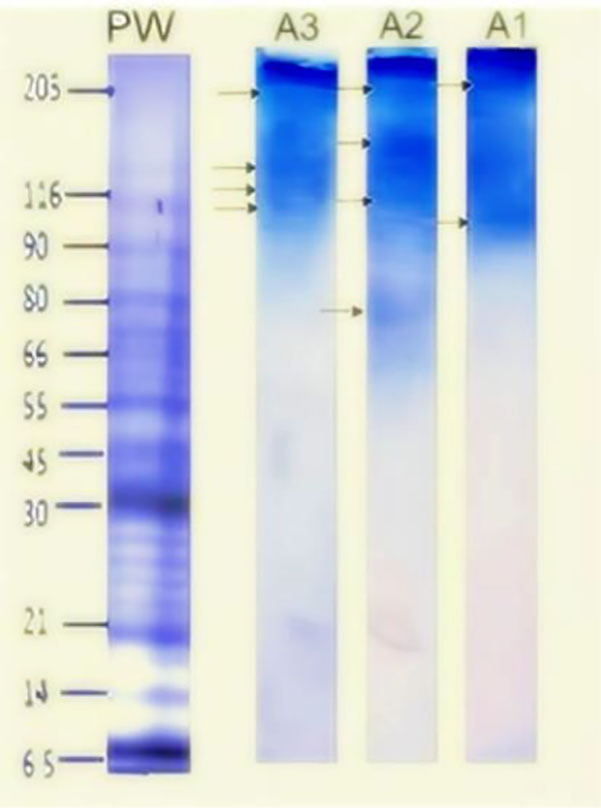
SDS‐PAGE showing the protein profile in Beers A1, A2, and A3. PW on the left refers to the molecular weight marker.

To assess the impact of protease addition on the protein profile of the produced beers, SDS‐PAGE electrophoresis was conducted. The results are illustrated in Figure 1. All three treatments displayed distinct bands, indicating a significant concentration of proteins in the evaluated beers.

After protease treatment, the protein profile of Beer A2 was different from those of Beers A3 and A1, which were also treated with proteases. This suggests that the treatments caused changes in the protein profile.

Sample A2 shows distinct bands in the high molecular weight range (from 70 to 205 kDa), indicating the presence and abundance of larger or complex proteins naturally found in barley‐based beers, such as hordeins, lipid transfer proteins (LTP1), protein Z, and albumins (Cramer et al. 2024). Similarly, formulation A1 also displays bands in the high molecular weight region, further suggesting the presence of larger and complex proteins. This implies a lower abundance, likely due to partial degradation caused by the added enzymes. In comparison, formulation A2 exhibits a more noticeable reduction in high molecular weight proteins than A3 and A1, whereas A1 still shows a residue in the 100 kDa range.

The results preliminarily indicate that protease treatments in Samples A1 and A2 influenced protein degradation. Interestingly, differences were observed in the recorded bands of A2 and A1, suggesting that the addition of proteases during fermentation or maturation led to variations in the resulting protein profile when compared to each other or A3.

In studies focused on low‐gluten beer production with the addition of AN‐PEP during fermentation, less intense bands were noted in the low‐molecular‐weight regions as the enzyme concentration increased. This finding supports the results of our study (Fiedler et al. 2019). Furthermore, research conducted by Liu et al. (2023) using the AN‐PEP enzyme under similar conditions achieved gluten levels below the detection limit of 10 mg/kg. These results, along with our findings, suggest that the enzyme used contributed to the reduction of gluten in the beers examined.

### 3.4 Analysis of Volatile Compounds

#### 3.4.1 Selection of SPME Fiber Coating Material

**FIGURE 2:**
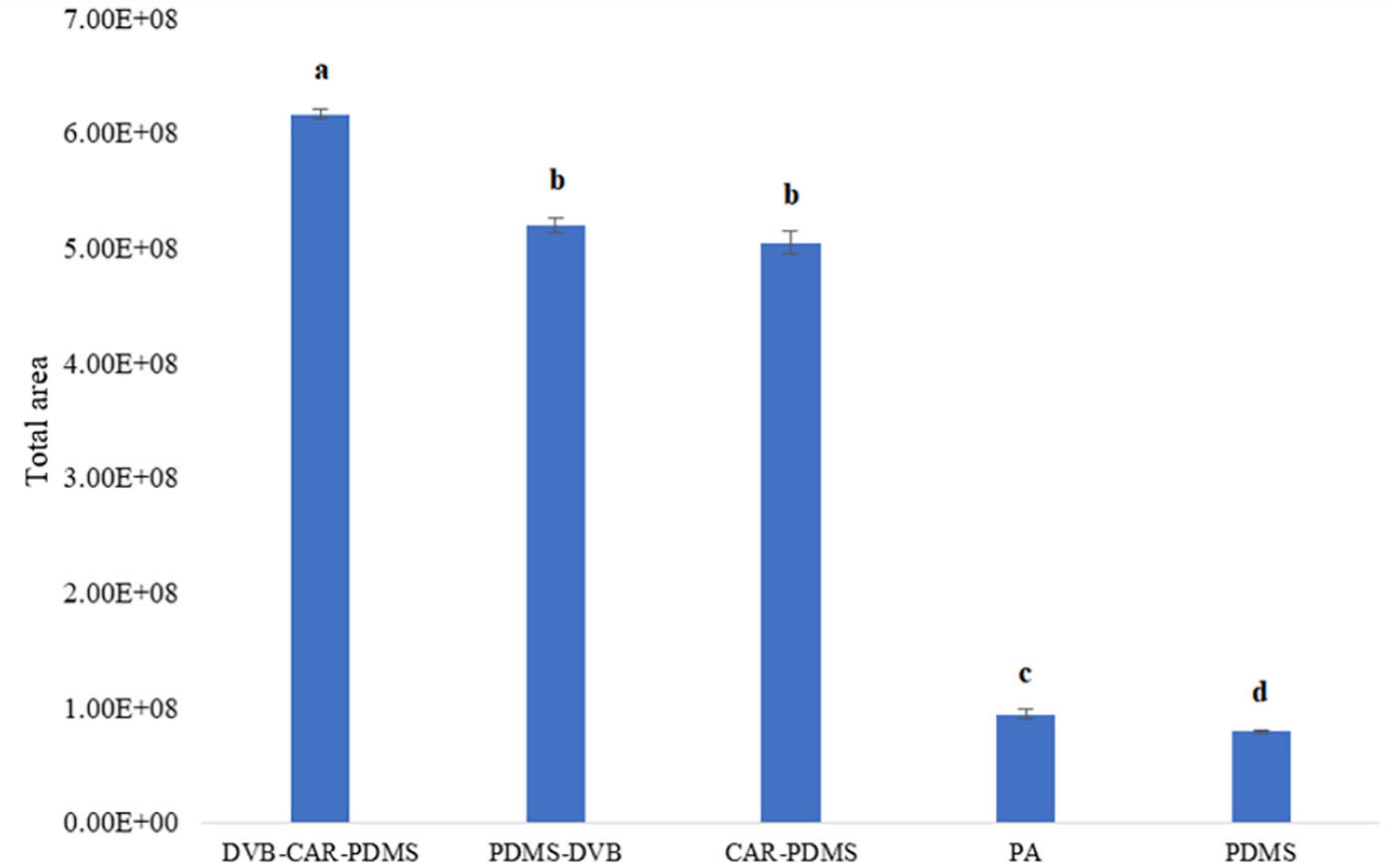
Extraction efficiency of the tested SPME fibers for volatile capture of a new fruit beer product with reduced gluten content, cupuassu, by HS‐SPME. The result is expressed as the mean of triplicate measurements for total fiber area obtained by GC–MS. Different lower letters in the bars indicate statistically significant differences by Tukey test at a 95% confidence level.

Figure 2 displays the results for the total area obtained from each of the five SPME fibers used in the test. The fiber that yielded the largest total area was the DVB/CAR/PDMS blend, followed by the PDMS/DVB and CAR/PDMS fibers. Numerous studies have indicated that the DVB/CAR/PDMS fiber is the most effective for volatile analysis in food (Bertolo et al. 2025; Balthazar et al. 2024; Spada et al. 2024; Almeida et al. 2023). Consequently, this fiber was chosen for the study on optimizing volatile extraction.

#### 3.4.2 Optimization of Volatile Extraction by SPME

The results, expressed as the total area of the chromatograms, from the central composite rotational design used to identify the optimal volatile extraction conditions in the tested formulations through HS‐SPME–GC–MS with the DVB/CAR/PDMS fiber, showed that the highest values were recorded in experiment Number 4, which reached 1.36 × 10^12^. This was achieved by extracting for 30 min at 35°C. The second highest value was observed in experiment Number 8, with a total area of 1.19 × 10^12^, obtained by extracting for 32 min at 28°C (Table 4).

**TABLE 4:** Experimental conditions and total area values of the chromatograms obtained in the optimization stage of the extraction conditions by SPME of the volatiles in the formulations investigated by headspace solid‐phase microextraction (HS‐SPME).

Experiment number	X1 (°C)	Temperature (°C)	X2 (min)	Time (min)	Total area^a^
1	−1	20	−1	20	4.366 × 10^11^
2	1	35	−1	20	8.594 × 10^11^
3	−1	20	1	30	6.779 × 10^11^
4	1	35	1	30	1.362 × 10^12^
5	−1.41	17	0	25	4.771 × 10^11^
6	1.41	38	0	25	1.107 × 10^12^
7	0	28	−1.41	18	5.171 × 10^11^
8	0	28	1.41	32	1.198 × 10^12^
9	0	28	0	25	8.938 × 10^11^
10	0	28	0	25	1.071 × 10^12^
11	0	28	0	25	9.33 × 10^11^
12	0	28	0	25	1.007 × 10^12^

^a^ Total area of the GC–MS chromatogram, expressed in arbitrary units.

**FIGURE 3:**
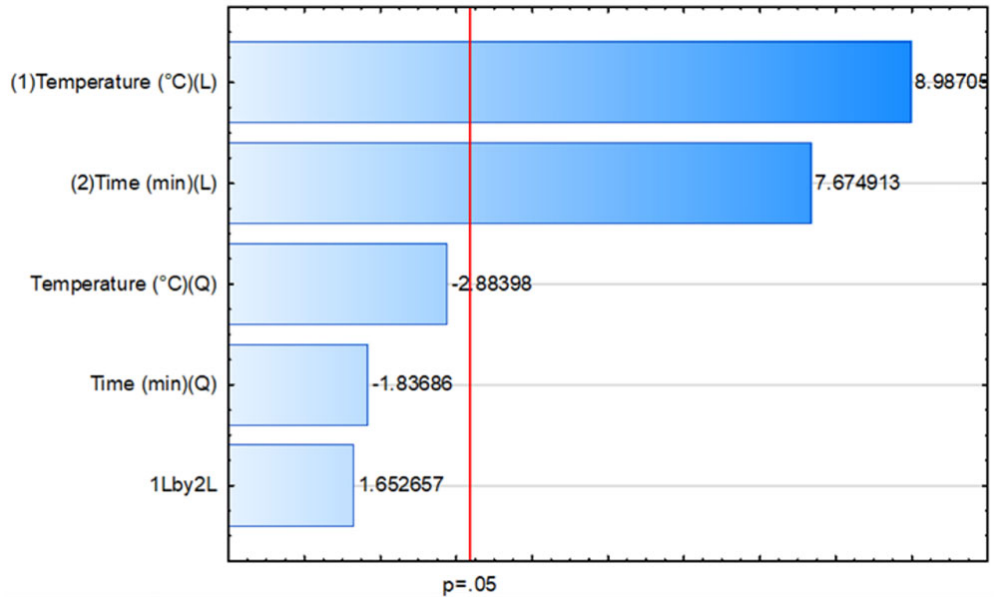
Pareto diagram of the effects of the extraction time and temperature variables studied in the optimization stage of the SPME extraction conditions of volatiles in the investigated formulations. Answer: total area of the GC–MS chromatograms, two factors, one block, 12 experiments, pure error 6.19E + 21.

Figure 3 shows the Pareto diagram, which summarizes the experimental results obtained using DCCR with DVB/CAR/PDMS fiber. The parameters linear extraction temperature (*L*) and linear extraction time (*L*) were significant at the 95% confidence interval (*p* = 0.05). The quadratic extraction temperature (*Q*), quadratic extraction time (*Q*), and the interaction between temperature and extraction time were not significant (Zacaroni et al. 2017; Šikuten et al. 2021; Zhang et al. 2021).

**FIGURE 4:**
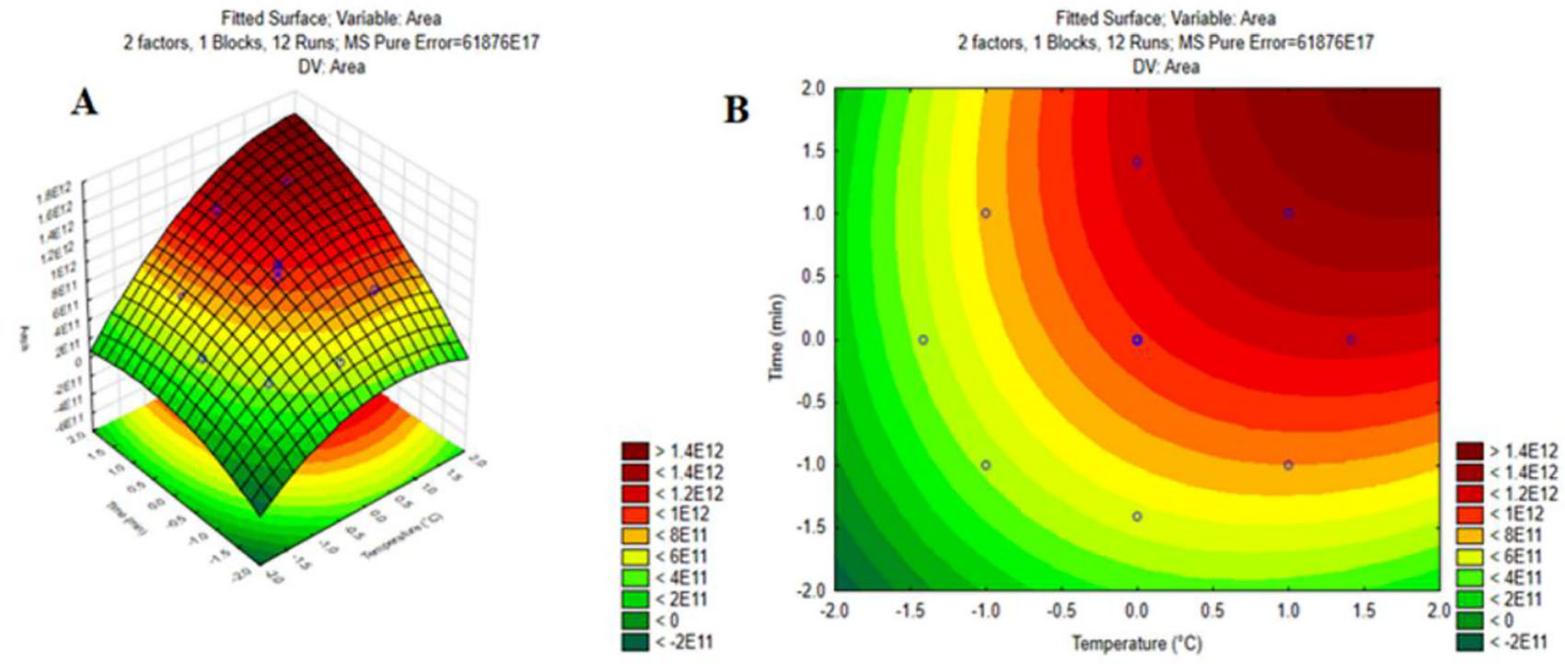
Response surface obtained by the quadratic model for the total area response of the chromatogram *z* = 9.76E + 11 + 2.50E + 11*x* − 9.01E + 10*x* ^2^ + 2.14E + 11*y − *5.71E + 10*y* ^2^ + 6.53E + 10*xy* (Figure 4A), and contour plot of the optimization of the extraction temperature conditions (*T*, °C) and extraction time (*t*, min) of the volatiles in the investigated formulations, by HS‐SPME (Figure 4B).

Figure 4 shows the response surface (Figure 4A) and contour plot (Figure 4B) obtained for the complete area response in the DCCR. A region of maximum analytical signal can be observed in the red regions (area > 1.40 × 10^12^), corresponding to the values of 35°C and 30 min for the extraction temperature and time, respectively.

#### 3.4.3 Volatile Profile of Investigated Beers

The previously optimized extraction conditions were applied to analyze the samples. Table 5 presents the identified compounds in the investigated samples, along with their mean gross area values (multiplied by 10^−6^) and corresponding odor descriptors. It is important to note that the area values represent relative measurements and do not indicate the actual amounts of volatiles, as they are not quantitative data. In this research, we utilized these parameters to compare the variations in volatile compounds across the samples.

**TABLE 5:** Volatile compounds identified by HS‐SPME and GC–MS on the three investigated formulations (A1, A2, and A3) with their belonging chemical class and respective odor description.

Chemical class	Compound	RI cal.	RI lit.	Δ	A1^a^	A2^a^	A3^a^	Odor description
**Esters**								
	Ethyl propionate	736	726	10	0.37	0.12	0.27	Fruity
	Ethyl isobutyrate	766	762	4	0.52	0.14	0.62	Fruity, strawberry, sweet
	Isobutyl acetate	778	779	−1	0.97	0.24	1.73	Fruity, apple, banana
	Ethyl butyrate	799	800	−1	10.34	4.89	37.83	Fruity, apple
	Butyl acetate	812	814	−2	1.04	0.59	2.47	Fruity pear
	Ethyl 2‐methylbutyrate	846	848	−2	3.96	1.38	9.4	Fruity, apple
	Isopentyl acetate	876	876	0	13.58	1.74	11.42	Fruity, banana, pear, sweet, fresh
	2‐Methylbutyl acetate	885	886	−1	3.94	1.38	2.79	Fruity
	Butyl‐isobutyrate	951	955	−4	0.4	0.23	1.29	Sweet, fruity, tropical, green, apple
	Ethyl hexanoate	999	999	0	17.43	4.4	25.87	Fruity, apple peel
	Isopentyl isobutyrate	1012	1013	−1	0.12	0.07	0.53	Fruity, apricot, pineapple, banana, sweet
	2‐Methylbutyl isobutyrate	1015	1014	1	0.44	0.09	1.06	Fruity, ethereal, tropical, banana
	Butyl 2‐methylbutanoate	1041	1043	−2	4.46	1.39	8.21	Fruity, green, cocoa, sweet
	Isoamyl butyrate	1055	1054	1	0.3	0.24	1.57	Sweet, apricot, banana
	2‐Methylbutyl 3‐methylbutanoate	1141	1147	−6	0.15	0.08	0.65	Flowery, blackberry peely, light tobacco
	Ethyl octanoate	1197	1197	0	18.21	4.02	5.47	Fruity, fatty, floral, green
	Phenethyl acetate	1258	1258	0	4.31	0.74	2.68	Rose, honey, tobacco
	Isobornyl acetate	1289	1287	2	0.49	0.33	0.23	Balsamic, camphor, herbal, woody, sweet
	Methyl geraniate	1326	1322	4	0.65	0.15	0.12	Flower, green, fruit
	Citronellyl acetate	1354	1354	0	0.36	0.43	0.43	Rose, dust
	Ethyl decanoate	1395	1395	0	3.61	0.6	0.87	Grape, fruity
**Alcohols**								
	3‐Methyl‐1‐butanol	749	750	−1	95.53	35.44	121.19	Whiskey, malt, burnt
	2‐Methyl‐1‐butanol	754	753	1	33.72	11.34	25.48	Malty
	2,3‐Butanediol	787	782	5	0.29	0.06	1.22	Fruity, creamy, buttery
	1‐Hexanol	867	867	0	3.05	1.47	4.58	Flowery, resin, green
	1‐Heptanol	971	971	0	0.61	0.07	0.93	Mushroom
	2‐Octen‐1‐ol	1068	1066	2	1.75	0.72	0.19	Soapy, green, vegetable
	1‐Octanol	1072	1072	0	1.37	0.63	0.83	Floral, chemical, metal, burnt
	1‐Phenyl‐1‐propanol	1097	—	—	0.49	0.1	0.54	Balsamic, floral, sweet
	Linalool	1099	1100	−1	80.47	42.27	136.85	Flowery, lavender
	2‐Phenylethanol	1113	1114	−1	60.14	31.03	35.77	Honey, sweet, floral, rose
	Nonanol	1173	1172	1	1.69	0.21	0.84	Faty, green, fruity
	Alpha‐terpineol	1192	1191	1	3.76	1.25	4.29	Pine, lilac, citrus, woody, floral
	Citronellol	1229	1229	0	12.07	4.57	8.73	Rose, sweet, citrus
	2‐*Tert*‐butylcyclohexan‐1‐ol	1295	1301	−6	0.37	0.36	0.34	Herbal, pine, camphor, minty, patchouli
**Carboxylic acids**								
	Butyric acid	995	996	−1	1.76	1.41	7.75	Rancid, cheese, sweat
	Octanoic acid	1186	1186	0	4.13	0.34	0.88	Sweat, cheese
	9‐Decenoic acid	1388	1389	−1	1.52	0.33	0.6	Waxy, green, fatty, soapy
**Aldehydes**								
	Octanal	1002	1003	−1	6.66	3.83	7.57	Faty, soapy, lemon, green
	Nonanal	1104	1104	0	16.92	7.36	0.65	Fatty, citrus, green
	Decanal	1206	1206	0	21.18	7.03	12.21	Soap, orange peel, tallow
	Undecanal	1307	1303	4	1.04	0.34	0.48	Fruity, green, waxy, oily
	Dodecanal	1410	1409	1	0.62	0.18	0.3	Herbal, fatty, citrus, waxy
**Ketone**								
	Sulcatone	986	987	−1	1.39	0.41	1.43	Fruity, apple, musty, ketonic, and creamy
	Fenchone	1088	1088	0	4.86	2.37	15.61	Camphor
**Terpenes**								
	Beta‐myrcene	991	990	1	3.45	1.45	3.8	Balsamic, must, spice
	Limonene	1029	1028	1	1.51	0.88	2.48	Citrus, lemon, orange
	Beta‐ocimene	1038	1032	6	0.19	0.09	0.33	Citrus, herb, flower
	*Trans*‐beta‐ocimene	1048	1048	0	0.35	0.07	0.75	Herbal, mild, citrus, sweet, orange, lemon
	Alpha‐terpinyl methyl ether	1157	—	—	0.88	0.15	0.61	Citrus, grapefruit, bergamot, orangeflower
**Sesquiterpenes**								
	Alpha‐copaene	1381	1382	−1	0.45	0.06	0.59	Woody, spice
	Caryophyllene	1425	1423	2	5.3	0.6	0.71	Woody, spicy
	Aromandendrene	1434	1439	−5	0.26	0.03	1.69	Sweet, dry
	*Trans*‐α‐bergamotene	1439	1434	5	0.35	0.05	0.13	Warm, tea leaf
	Humulene	1460	1460	0	1.45	1.6	0.17	Woody
	Valencene	1481	1480	1	1.01	0.11	0.23	Pepper, orange
	Beta‐selinene	1492	1492	0	1.19	0.13	0.32	Herbal
	Gamma‐cadinene	1520	1521	−1	0.62	0.06	0.09	Herbal
	Delta‐cadinene	1528	1528	0	0.95	0.12	0.22	Herbal
	Alloaromadendrene oxide	1641	1641	0	0.4	0.19	0.15	—

*Note*: The area values, presented for the analytes, are the gross area of the chromatogram multiplied by 10^−6^. RI cal.: experimentally obtained retention index (calculated); RI lit.: retention index obtained from the literature; Δ = difference between the theoretical and calculated retention indices (RI lit.—RI cal.).

^a^ The area values, presented for the analytes, are the raw chromatogram area multiplied by 10^−6^.

A total of 60 volatile compounds (Table 5) were identified in the samples. Among these, 35% (21 compounds) belonged to the ester class, 23% (14) were classified as alcohols, 16% (10) as sesquiterpenes, 8% (5) as terpenes, and 16% (10) as aldehydes, ketones, and acids.

The compounds found to have the highest intensity in the evaluated beers were 3‐methyl‐1‐butanol, which is characterized by a whiskey, malt, and burnt aroma. This was followed by linalool, known for its flowery aroma, and 2‐phenylethanol, which has a honey‐like, sweet, floral, and rose aroma.

It was noted that considerable quantities of esters, carboxylic acids, alcohols, aldehydes, ketones, terpenes, and pyrazines are present. Research on the volatile compounds in the pulp of *T. grandiflorum* revealed that compounds, such as ethyl butanoate, ethyl hexanoate, and linalool, were predominant, which explains the high linalool content in the analyzed beers (Quijano and Pino 2007).

**FIGURE 5:**
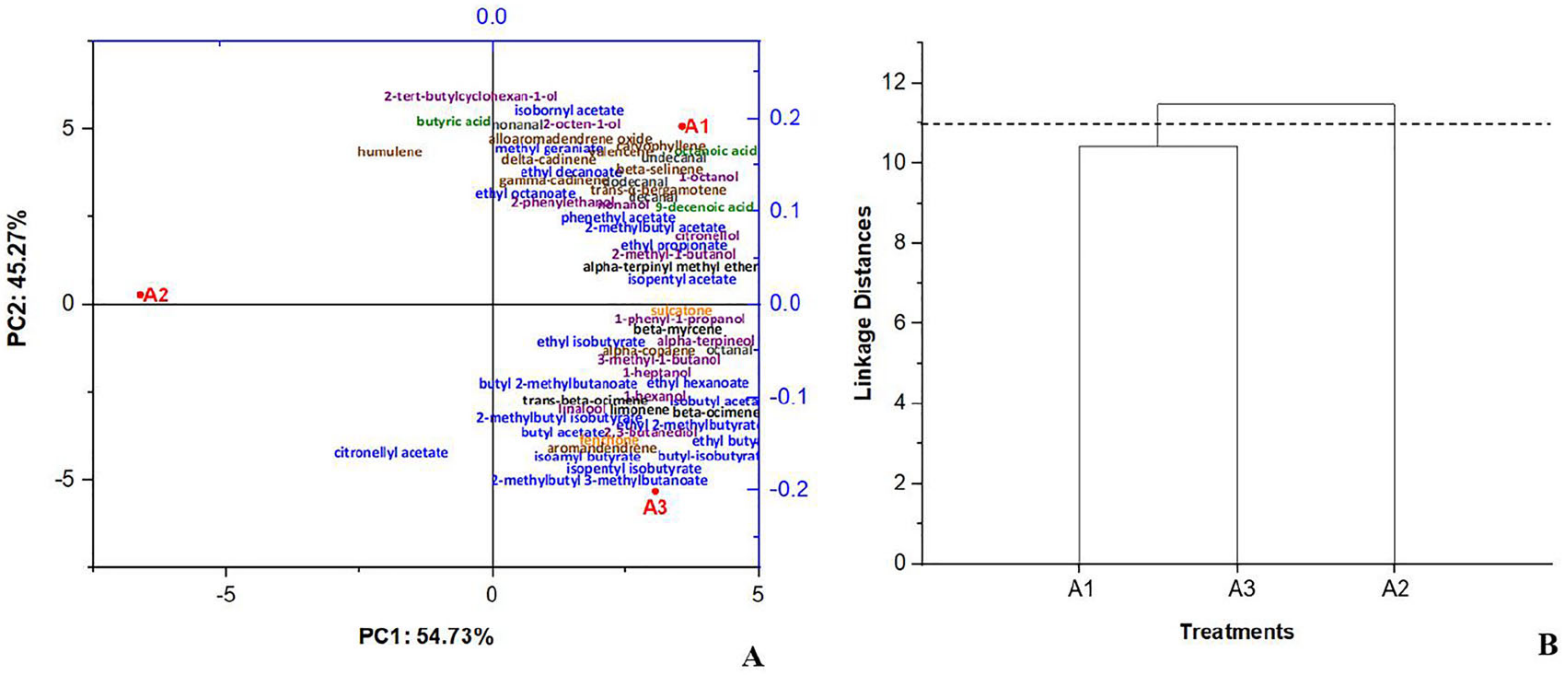
Profile of principal components analysis (A) and hierarchical cluster analysis (B) of investigated beers related to volatile compounds distribution.

The analysis using Mahalanobis distance confirmed the high concentrations of three compounds: 3‐methyl‐1‐butanol, linalool, and 2‐phenylethanol. These compounds exhibited significantly different behaviors compared to other volatile compounds, as evidenced by their Mahalanobis distances exceeding the threshold of 2.881 (Figure 5). The presence of 3‐methyl‐1‐butanol, linalool, and 2‐phenylethanol is crucial to the sensory profile of the beers examined, according to the Mahalanobis distance analysis. The higher concentration of linalool in the studied beers can be attributed to its status as a major component of hop essential oils. Linalool concentrations ranged from 62.3 ± 4.7 to 155 ± 8.0, which aligns closely with the values reported in a previous study by Schmidt and Biendl (2016).

**FIGURE 6:**
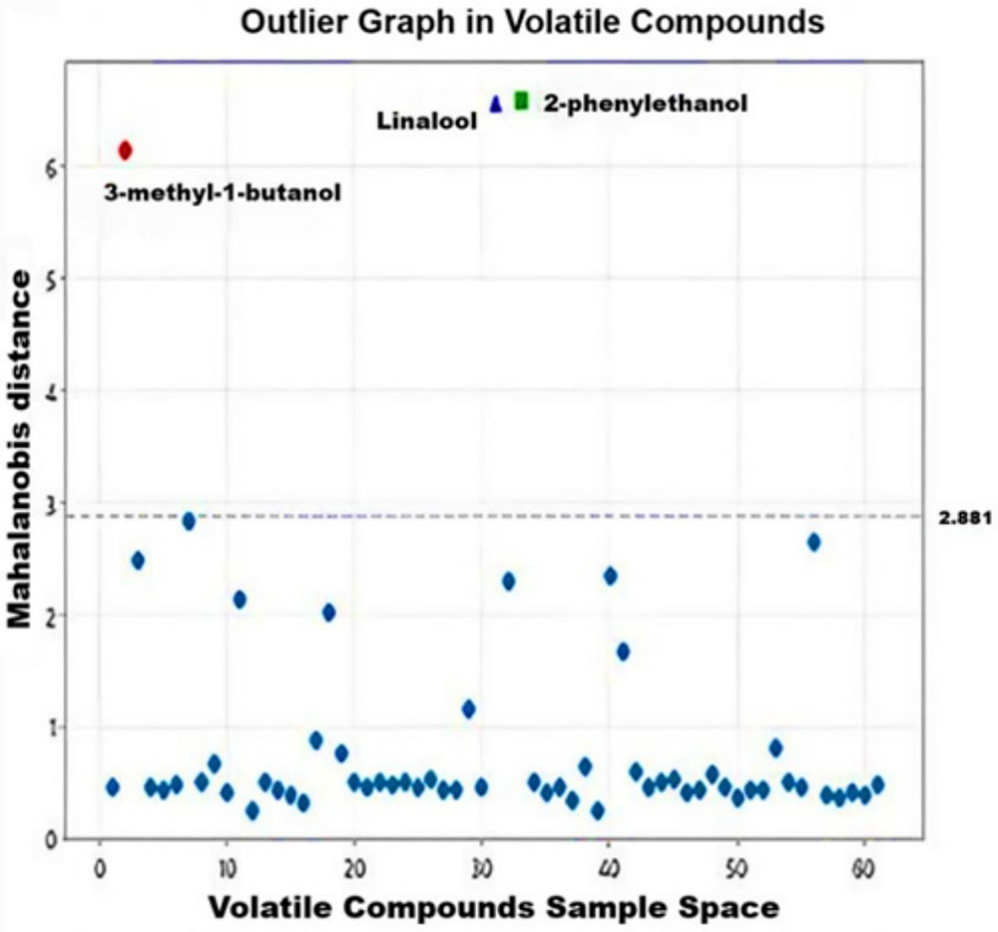
Analysis of volatile compounds in beers using Mahalanobis distance analysis. As illustrated in Figure 5A, sample A3 has a higher concentration of linalool because it is located in the same quadrant, whereas 2‐phenylethanol is found in greater quantities in Sample A1. These findings suggest a predominance of an aromatic profile characterized as floral, sweet, and whiskey‐like in the formulated beers.

The observed concentrations of higher alcohols can be explained mechanistically by AN‐PEP proteolysis. This enzyme hydrolyzes peptide bonds in high‐molecular‐weight malt proteins, generating smaller peptides and free amino acids (Bretträger et al. 2023). These compounds act as precursors in the Ehrlich pathway of yeasts, being transaminated and decarboxylated to form the respective fusel alcohols (3‐methyl‐1‐butanol from leucine and 2‐phenylethanol from phenylalanine) (Dzialo et al. 2017). The early addition of the enzyme during primary fermentation (Treatment A1) increased the availability of assimilable nitrogen throughout the fermentation, resulting in greater apparent attenuation and higher production of these alcohols, with a positive sensory impact (notes of whiskey/burnt malt and honey/rose/floral). In Treatments A2 and A3, the lower availability or the absence of this proteolytic release reduced the formation of these compounds, as evidenced in the data in Table 5 and in the multivariate analyses (Figures 5 and 6). Thus, AN‐PEP acts not only in the potential reduction of immunoreactive fractions of gluten (Cela et al. 2020; Liu et al. 2023) but also as a positive modulator of the volatile profile of beer, synergistically enhancing the floral and fruity notes already inherent in cupuassu pulp, rich in linalool and esters (Quijano and Pino 2007) (Table 6).

**TABLE 6:** Loadings of contributing volatile compounds (VOCs) for each PC in descending order.

Volatile compounds	CP1	Volatile compounds	CP2
Isopentyl acetate	0.172	Isoamyl butyrate	0.177
nonanal_1	0.172	butyric acid	0.176
Sulcatone	0.169	Isopentyl isobutyrate	0.172
2‐Methyl‐1‐butanol	0.168	2‐Methylbutyl 3‐methylbutanoate	0.17
Alpha‐terpinyl methyl ether	0.168	Aromandendrene	0.169
Ethyl propionate	0.167	Ethyl butyrate	0.167
1‐Phenyl‐1‐propanol	0.166	Butyl‐isobutyrate	0.167
2‐Methylbutyl acetate	0.164	Fenchone	0.165
Beta‐myrcene	0.164	2.3‐Butanediol	0.164
Citronellol	0.164	Butyl‐acetate	0.159
Phenethyl acetate	0.164	Ethyl 2‐methylbutyrate	0.149
Alpha‐terpineol	0.163	Citronellyl acetate	0.147
Ethyl isobutyrate	0.161	2‐Methylbutyl isobutyrate	0.143
Octanal	0.159	Limonene	0.138
Alpha‐copaene	0.157	Beta‐ocimene	0.138
Nonanol	0.155	Linalol	0.137
3‐Methyl‐1‐butanol	0.154	*Trans*‐beta‐ocimene	0.136
Decanal	0.15	Butyl 2‐methylbutanoate	0.13
1‐Heptanol	0.148	Isobutyl acetate	0.123
Ethyl hexanoate	0.145	1‐Hexanol	0.12
Dodecanal	0.14	Ethyl hexanoate	0.102
*Trans*‐α‐bergamotene	0.14	1‐Heptanol	0.098
1‐Octanol	0.139	3‐Methyl‐1‐butanol	0.085
9‐Decenoic acid	0.135	Alpha‐copaene	0.079
1‐Hexanol	0.134	Octanal	0.074
Undecanal	0.133	Ethyl isobutyrate	0.068
Isobutyl acetate	0.132	Alpha‐terpineol	0.061
Beta‐selinene	0.13	Beta‐myrcene	0.057
2‐Phenylethanol	0.128	1‐Phenyl‐1‐propanol	0.052
Butyl 2‐methylbutanoate	0.127	Sulcatone	0.037
Octanoic acid	0.126	nonanal_1	0.001
Valencene	0.124	Isopentyl acetate	−0.002
Delta‐cadinene	0.123	2‐Methyl‐1‐butanol	−0.04
*Trans*‐beta‐ocimene	0.121	Alpha‐terpinyl methyl ether	−0.04
Ethyl octanoate	0.121	Ethyl propionate	−0.046
Linalool	0.12	Citronellol	−0.056
Limonene	0.119	2‐Methylbutyl acetate	−0.057
Beta‐ocimene	0.119	Phenethyl acetate	−0.058
Ethyl decanoate	0.119	Nonanol	−0.084
Gamma‐cadinene	0.116	Decanal	−0.094
2‐Methylbutyl isobutyrate	0.115	Dodecanal	−0.111
Caryophyllene	0.112	*Trans*‐α‐bergamotene	−0.112
Ethyl 2‐methylbutyrate	0.108	1‐Octanol	−0.113
Methyl geraniate	0.102	9‐Decenoic acid	−0.119
Butyl‐acetate	0.097	Undecanal	−0.121
Humulene	0.096	Beta‐selinene	−0.126
2.3‐Butanediol	0.09	2‐Phenylethanol	−0.128
Alloaromadendrene oxide	0.09	Octanoic acid	−0.131
Fenchone	0.088	Valencene	−0.132
Ethyl butyrate	0.085	Delta‐cadinene	−0.134
Butyl‐isobutyrate	0.084	Ethyl octanoate	−0.136
Aromandendrene	0.081	Ethyl decanoate	−0.138
2‐Methylbutyl 3‐methylbutanoate	0.079	Gamma‐cadinene	−0.141
Isopentyl isobutyrate	0.076	Caryophyllene	−0.146
Butyric acid	0.069	Methyl geraniate	−0.154
Isoamyl butyrate	0.066	Humulene	−0.159
2‐Octen‐1‐ol	0.058	Alloaromadendrene oxide	−0.164
Isobornyl acetate	0.048	2‐Octen‐1‐ol	−0.181
Nonanal	0.045	Isobornyl acetate	−0.184
2‐*Tert*‐butylcyclohexan‐1‐ol	0.013	Nonanal	−0.185
Citronellyl acetate	−0.11	2‐*Tert*‐butylcyclohexan‐1‐ol	−0.191

### 3.5 Multivariate Analysis (PCA and HCA) of Beers

PCA was conducted on 60 variables (volatile compounds), resulting in two principal components that together (PC1 + PC2) explained 100% of the total variance. This analysis provided valuable insights into the distribution of volatiles in the three beers investigated.

Figure 5, PC1, responsible for 54.73% of the variance, separated A1 and A3 on the positive axis and isolated A2 at the negative extreme, indicating that the latter presents a significantly distinct and less complex aromatic profile. This separation is strongly influenced by the high contribution of citronellyl acetate, which acts as a determining chemical marker and explains the isolated position of A2 in relation to the other samples. This behavior suggests relevant differences in the formulation, yeast strain, or fermentation conditions of this sample compared to the multivariate pattern observed in the other two beers.

PC2, which represents 45.27% of the variance, further differentiated A1 and A3, revealing a chemical gradient that contrasts heavy compounds, such as higher alcohols and medium‐chain esters (associated with A1), with lighter and fruitier compounds characteristic of A3. A1 exhibits a prevalence of metabolites linked to intense fermentation pathways, conferring a denser aromatic profile, whereas A3 aligns with compounds of greater freshness and lower molecular weight. The combined configuration of the two components shows that the samples diverge from each other and express volatile signatures consistent with distinct technological processes, reinforcing the ability of PCA to capture metabolic and sensory nuances relevant to the characterization and differentiation of beers.

Figure 6, which shows the Mahalanobis distance, includes the compounds 3‐methyl‐1‐butanol (represented by a red dot), linalool (indicated by a blue triangle), and 2‐phenylethanol (indicated by a green square). The distinct distance between these groups confirms their higher concentration in beer samples. A descriptive analysis of the loadings by principal component, showing the behavior of the volatile compounds, is presented in Table S3.

To categorize the volatile compound profile data in the investigated beers, HCA was applied. Figure 6B presents a dendrogram with the similarity measures between the clusters, represent Beers A1, A2, and A3. HCA analysis indicates that Beers A1 and A3 have similar quantitative volatile profiles, whereas Beer A2 showed significant differences. A descriptive analysis of the volatile compounds, including their mean concentrations (centroids) for the two groups identified by HCA, is presented in Table S3.

### 3.6 Practical Applications and Industrial Relevance

The study offers immediate practical application to the craft and industrial brewing sector: (i) It allows the production of low‐alcohol fruit beers (2.8%–3.4% ABV) with an intense floral profile, sweetness, and whiskey notes; (ii) it enhances the value of cupuassu, an Amazonian fruit, giving it a regional identity; (iii) it demonstrates that a food‐grade enzyme (AN‐PEP) added during fermentation or maturation can preliminarily hydrolyze high molecular weight proteins; and (iv) it is fully compatible with existing production processes, without the need for additional equipment.

## 4 Conclusion

The addition of cupuassu pulp proved viable in the production of fruit beers, characterized by a predominantly floral, sweet, and whiskey‐like aromatic profile, which is justified by the high content of 3‐methyl‐1‐butanol, linalool, and 2‐phenylethanol. Thus, we can observe that the use of cupuassu as an adjunct in craft beers presents pleasant characteristics, making its application favorable for fruit beer production.

The applied manufacturing technology also enabled the production of low‐alcohol beers, which may represent a valuable alternative for consumers who prefer this segment of beers.

The addition of the AN‐PEP protease demonstrated the ability to degrade high‐molecular‐weight proteins, as evidenced by SDS‐PAGE analysis of the treated samples. However, in the absence of direct immunological quantification of gluten—such as by competitive ELISA R5 or another validated method—it is not possible to determine the residual gluten content or to confirm that the beers meet the regulatory criteria for “very low gluten” (<100 mg/kg) or “gluten free” (<20 mg/kg). Such immunological analysis was not performed in the present study and remains an essential requirement for future work to enable accurate classification of the beers according to established regulatory limits.

Further research is needed, as this study did not investigate the gluten profile of the beers studied but only the preliminary effect of the enzyme's action on the protein profile based on the estimation of their molecular weights. Although the floral, sweet, and whiskey‐like aromatic profile was inferred from the identification and relative quantification of the major volatile compounds (3‐methyl‐1‐butanol, linalool, and 2‐phenylethanol) and their recognized olfactory descriptors, no formal sensory evaluation (trained panel or affective test with consumers) was performed. Such an analysis would be essential to confirm consumers’ actual perception of these attributes and validate the sensory claims presented. This constitutes an important limitation of the present work and will be the subject of investigation in future studies.

## Author Contributions

**Ermor Cesar de Sousa Lopes**: conceptualization, investigation, writing – original draft, methodology, visualization, formal analysis, data curation. **Nélio Jacinto Manuel Ualema**: investigation, writing – review and editing. **Hellen Kempfer Philippsen**: conceptualization, investigation, visualization, writing – review and editing. **Camilo Barroso Teixeira**: conceptualization, investigation, writing – review and editing. **Stanislau Bogusz Junior**: conceptualization, investigation, visualization, validation, formal analysis, writing – review and editing, data curation. **Guilherme Ribeiro da Cunha Nascimento**: formal analysis, investigation, conceptualization, data curation. **Gilson C. A. Chagas‐Junior**: visualization, writing – review and editing, data curation. **Braian Saimon Frota da Silva**: data curation, formal analysis, investigation, writing – review and editing. **Nelson Rosa Ferreira**: conceptualization, investigation, funding acquisition, writing – review and editing, methodology, project administration, supervision, resources, data curation.

## Conflicts of Interest

The authors declare no conflicts of interest.

## Supporting information




**Supplementary Material**: jfds70916‐sup‐0001‐SuppMat.docx

## Data Availability

The authors have nothing to report.
