# Supplementary material for: Production of Special Fruit Beer With Addition of Cupuassu (Theobroma grandiflorum) Pulp and Prolyl Endopeptidase to Improve Volatile Compounds and Physicochemical Parameters
Source: J Food Sci. 2026 Feb 19;91(2):e70916. doi: 10.1111/1750-3841.70916 (PMC12919682; doi:10.1111/1750-3841.70916)
Supplement: Supplementary file 1 — Supplementary Material: jfds70916‐sup‐0001‐SuppMat.docx [file JFDS-91-0-s001.docx]

Supplementary material, optimization method S1-A

**Optimization of sample preparation and selection of SPME fiber**

To optimize the sample preparation step for volatile analysis, 1000 µL aliquots of beer were transferred, using an adjustable-volume volumetric pipette, to 40 mL glass vials suitable for SPME extraction, equipped with a screw cap and PTFE/silicone septum. A magnetic stir bar and a microprocessor-controlled magnetic stirrer (IKA, model C-MAG HS 7, Staufen) were used to agitate the samples during extraction (IKA, C-MAG HS 7, Staufen im Breisgau, Germany) at 250 rpm. The vials containing the samples were further placed in a jacketed beaker, connected to a thermostated bath (Tecnal, model TE-184/1, Piracicaba, SP, Brazil) to control the temperature during extraction. In order to select the SPME fiber with the best extraction capacity of the volatiles from the samples, 5 different commercial SPME fiber coating materials were tested: polydimethylsiloxane (PMDS – 100 µm), polydimethylsiloxane / divinylbenzene (PMDS/DVB – 65 µm), polydimethylsiloxane / carboxen (CAR/PDMS – 75 µm), divinylbenzene / carboxen / polydimethylsiloxane (DVB/CAR/PDMS – 50/30 µm) and polyacrylate (PA – 58 µm). Before use, all fibers were pre-conditioned in the GC-MS injector according to the manufacturer's recommendations. For preliminary selection, all fibers were tested to select the one with the best sample volatile capture capacity. At this stage, all fibers were exposed to the sample headspace under the following extraction conditions: 20 min equilibration time, 30 min extraction time, and 30 °C extraction temperature. These conditions were arbitrarily established by the researchers only for the fiber selection stage. After extraction, the fibers were introduced into the GC-MS injector for desorption of the captured analytes at 250 °C in splitless mode for 1 min. The response evaluated at this stage was the total area of the chromatogram. After the extraction and desorption procedure, the fibers were reconditioned for 10 min at 250 °C. The fiber reconditioning procedure was performed to ensure that the fibers were clean for the next extraction. All fibers were tested in triplicate.

Supplementary material, optimization method S1-B

**Extraction optimization strategy​**

Once the fiber with the highest volatile extraction capacity was chosen, the extraction conditions were optimized through a central composite design (CCD), based on a factorial design 2^2^, with 4 axial points (α=1.41) and three repetitions at the central point (Bogusz et al., 2011). The optimized variables were extraction time (t, min) and extraction temperature (T, °C), the levels of each investigated variable can be seen in Table 2.

Supplementary material, table S1

Table S1 - Factors, levels and experimental domain of the conditions applied to optimize the extraction of volatiles from a new *fruit* beer produced with cupuassu with reduced gluten content, by HS-SPME .

| **Variables** | **Coded variables** | | | | |
| --- | --- | --- | --- | --- | --- |
|  | -α | -1 | 0 | 1 | α ^a^ |
| Extraction time (t. ext ., min) | 17 | 20 | 28 | 35 | 38 |
| Extraction temperature (T, °C) | 18 | 20 | 25 | 30 | 32 |

^a^ α = 1.41.

In total, twelve experiments were performed randomly, which are described in the following table (Table S2). The response evaluated at this stage was the total area of the chromatogram.

Supplementary material, table S2

Table S2 - Matrix of experiments that were carried out in the central composite design in the optimization stage of the extraction conditions of volatile compounds from a new *fruit* beer produced with cupuassu with reduced gluten content, by HS-SPME.

| **Rehearsal** | **Extraction time** | **Extraction temperature** |
| --- | --- | --- |
| 1 | -1 | -1 |
| 2 | 1 | -1 |
| 3 | -1 | 1 |
| 4 | 1 | 1 |
| 5 | -1.41 | 0 |
| 6 | 1.41 | 0 |
| 7 | 0 | -1.41 |
| 8 | 0 | 1.41 |
| 9 | 0 | 0 |
| 10 | 0 | 0 |
| 11 | 0 | 0 |
| 12 | 0 | 0 |

Supplementary material, table S3

Table S3 - Volatile compounds in order of importance according to their main components.

| **Volatile compounds** | **CP1** | **Volatile compounds** | **CP2** |
| --- | --- | --- | --- |
| isopentyl acetate | 0.172 | isoamyl butyrate | 0.177 |
| nonanal_1 | 0.172 | butyric acid | 0.176 |
| Sulcatone | 0.169 | isopentyl isobutyrate | 0.172 |
| 2-methyl-1-butanol | 0.168 | 2-methylbutyl 3-methylbutanoate | 0.17 |
| alpha-terpinyl methyl ether | 0.168 | aromandendrene | 0.169 |
| ethyl propionate | 0.167 | ethyl butyrate | 0.167 |
| 1-phenyl-1-propanol | 0.166 | butyl-isobutyrate | 0.167 |
| 2-methylbutyl acetate | 0.164 | fenchone | 0.165 |
| beta- myrcene | 0.164 | 2,3-butanediol | 0.164 |
| Citronellol | 0.164 | butyl acetate | 0.159 |
| phenethyl acetate | 0.164 | ethyl 2-methylbutyrate | 0.149 |
| alpha-terpineol | 0.163 | citronellyl acetate | 0.147 |
| ethyl isobutyrate | 0.161 | 2-methylbutyl isobutyrate | 0.143 |
| Octanal | 0.159 | limonene | 0.138 |
| alpha-copaene | 0.157 | beta- ocimene | 0.138 |
| Nonanol | 0.155 | linalool | 0.137 |
| 3-methyl-1-butanol | 0.154 | trans-beta- ocimene | 0.136 |
| Decanal | 0.15 | butyl 2-methylbutanoate | 0.13 |
| 1-heptanol | 0.148 | isobutyl acetate | 0.123 |
| ethyl hexanoate | 0.145 | 1-hexanol | 0.12 |
| Dodecanal | 0.14 | ethyl hexanoate | 0.102 |
| trans- *α* - bergamotene | 0.14 | 1-heptanol | 0.098 |
| 1-octanol | 0.139 | 3-methyl-1-butanol | 0.085 |
| 9-decenoic acid | 0.135 | alpha-copaene | 0.079 |
| 1-hexanol | 0.134 | octanal | 0.074 |
| Undecanal | 0.133 | ethyl isobutyrate | 0.068 |
| isobutyl acetate | 0.132 | alpha-terpineol | 0.061 |
| beta- selinene | 0.13 | beta- myrcene | 0.057 |
| 2-phenylethanol | 0.128 | 1-phenyl-1-propanol | 0.052 |
| butyl 2-methylbutanoate | 0.127 | sulcatone | 0.037 |
| octanoic acid | 0.126 | nonanal_1 | 0.001 |
| Valencene | 0.124 | isopentyl acetate | -0.002 |
| delta- cadinene | 0.123 | 2-methyl-1-butanol | -0.04 |
| trans-beta- ocimene | 0.121 | alpha-terpinyl methyl ether | -0.04 |
| ethyl octanoate | 0.121 | ethyl propionate | -0.046 |
| Linalool | 0.12 | citronellol | -0.056 |
| Limonene | 0.119 | 2-methylbutyl acetate | -0.057 |
| beta- ocimene | 0.119 | phenethyl acetate | -0.058 |
| ethyl decanoate | 0.119 | nonanol | -0.084 |
| gamma-cadinene | 0.116 | decanal | -0.094 |
| 2-methylbutyl isobutyrate | 0.115 | dodecanal | -0.111 |
| Caryophyllene | 0.112 | trans - β- bergamotene | -0.112 |
| ethyl 2-methylbutyrate | 0.108 | 1-octanol | -0.113 |
| methyl geraniate | 0.102 | 9-decenoic acid | -0.119 |
| butyl acetate | 0.097 | undecanal | -0.121 |
| Humulene | 0.096 | beta- selinene | -0.126 |
| 2,3-butanediol | 0.09 | 2-phenylethanol | -0.128 |
| alloaromadendrene oxide | 0.09 | octanoic acid | -0.131 |
| Fenchone | 0.088 | valencene | -0.132 |
| butyl-isobutyrate | 0.084 | ethyl octanoate | -0.136 |
| Aromandendrene | 0.081 | ethyl decanoate | -0.138 |
| 2-methylbutyl 3-methylbutanoate | 0.079 | gamma-cadinene | -0.141 |
| isopentyl isobutyrate | 0.076 | caryophyllene | -0.146 |
| butyric acid | 0.069 | methyl geraniate | -0.154 |
| isoamyl butyrate | 0.066 | humulene | -0.159 |
| 2-octen-1-ol | 0.058 | alloaromadendrene oxide | -0.164 |
| isobornyl acetate | 0.048 | 2-octen-1-ol | -0.181 |
| Nonannual | 0.045 | isobornyl acetate | -0.184 |
| 2-tert-butylcyclohexan-1-ol | 0.013 | nonanal | -0.185 |
| citronellyl acetate | -0.11 | 2-tert-butylcyclohexan-1-ol | -0.191 |
